# Which acupuncture and moxibustion technique is more effective for primary dysmenorrhea: A protocol for a network meta-analysis of randomized controlled trials

**DOI:** 10.1097/MD.0000000000021713

**Published:** 2020-08-28

**Authors:** Zenan Wu, Yi Yang, Jun Xiong, Xinyu Yu, Zhengyun Zuo, Qiongshan Xie

**Affiliations:** a Jiangxi University of Traditional Chinese Medicine; b The Affiliated Hospital of Jiangxi University of Traditional Chinese Medicine, Nanchang, China.

**Keywords:** acupuncture, moxibustion, primary dysmenorrhea, protocol, network meta-analysis, randomized controlled trials

## Abstract

**Background:**

Primary dysmenorrhea (PD), also called functional dysmenorrhea, refers to a woman's menstrual period in genital no organic disease, abdominal pain, under the belly and other discomfort for the characteristics of disease of department of gynecology. Acupuncture and moxibustion have been accepted as treatment options for PD. So far, there are so many therapies for PD and their efficacy has been assessed by several systematic reviews. Therefore, this study aims at evaluating the effectiveness which acupuncture and moxibustion technique is more effective for primary dysmenorrhea.

**Methods and analysis:**

The following electronic databases will be searched in this study: the Cochrane Central Register of Controlled Trials (CENTRAL);PubMed; EMBASE; China National Knowledge Infrastructure (CNKI); Chinese Biomedical Literature Database (CBM);Chinese Scientific Journal Database (VIP database); and Wan-Fang Database(WF). More than two authors independently assessed the quality of the evidence by AMSTAR2, PRISMA, PRISMA-A, and GRADE approach. Two of our researchers will use the bias risk tool provided by the Cochrane Collaboration to evaluate the quality of the literature using WinBUGS 1.4.3 and STATA softwares. The primary outcomes include the extent of pain in the lower abdomen measured by visual analog scale (VAS) and relief from symptoms. The quality of life (QoL) and Adverse events will be considered as Additional outcome(s). Their reference lists and the citation lists of studies meeting the inclusion criteria and relevant systematic reviews will also be searched to identify further studies for inclusion. Before this review completed, the 2 reviewers will conduct the search once again to ensure the latest studies could be included.

**Ethics and dissemination:**

This review does not require ethical approval.

**Results:**

The results will be published in a peer-reviewed journal.

**Conclusion:**

This study will provide comprehensive evidence of acupuncture and moxibustion for patients with PD.

**INPLASY registration number:**

INPLASY2020500106.

## 1 Introduction

### 1.1 Description of the condition

Primary dysmenorrhea (PD), as known as functional dysmenorrhea, is a common gynecological disease, it refers to spasmodic pain in the lower abdomen before, after or during menstruation, accompanied by general discomfort.^[1]^ According to a systematic review by the World Health Organization (WHO), the prevalence of dysmenorrhea in menstruating women is between 17% and 81%. Severe dysmenorrhea was identified in only 12% to 14% of cases.^[2]^ There is still little evidence on which Nonsteroidal anti-inflammatory drugs (NSAIDs) is more effective or safer, but, currently, the most widely used ones are ibuprofen, naproxen, mefenamic acid, and ketoprofen.^[3–7]^ Because they cause serious adverse effects, they are no longer used for the treatment of PD.^[8,9,10]^ The mechanism of acupuncture involves stimulation of nerve fibers and receptors in a complex interaction with endorphins and serotonins.^[11]^ A more recent randomized controlled trial (RCT) from Germany that examined 649 women found significant improvement in quality of life and decreased intensity of pain in the acupuncture-treated group relative to controls.^[12]^ Moxibustion is able to relieve menstrual pain and other discomfort of PD patients in various pass ways, and there are lots of verifying study results to confirm the effects and safety of moxibustion for PD.^[13,14,15]^ PD causes high rates of school and work absenteeism, as well as decreasing quality of life.^[16,17]^ In a study conducted in Portugal, 8.1% of girls reported missing school or work due to menstrual pain, impacting daily activities in 65.7% of cases. Only 27.9% sought medical help.^[18]^

However, there are many techniques can be used for the treatment of PD, but no study aims to evaluate the current evidence for the efficacy and safety of acupuncture and moxibustion therapies for the PD. Therefore, we decide to conduct a network meta-analysis of acupuncture and moxibustion for PD to collect some reliable evidence for clinical guidance and to assistant PD females to seek more reasonable treatments. In this study, we aim to evaluate the effectiveness which acupuncture and moxibustion technique is more effective for PD.

### 1.2 Description of the intervention

Acupuncture has been the oldest therapeutic method in the history of Traditional Chinese Medicine (TCM). The practice of acupuncture predates other ancient Chinese remedies, such as herbal medicines.^[19]^ Acupuncture has its roots in the early experience of injuries in humans, where placing sharp stones in specific positions on the body can relieve symptoms. The “Bian Stone” was the first stone needle used in acupuncture therapy. In just a few decades, acupuncture has become the most popular and widely recognized Chinese medicine therapy in the Western world,^[20,21]^ for example, acupuncture is widely used in chronic pain management. Some study showed that, both perpendicular and transverse needling at acupoint (SP6) had an immediate analgesic effect on pain caused by PD.^[22]^

Moxibustion therapy for PD is a family therapy with a long history. It mainly uses the heat generated by moxibustion to directly act on the lower abdomen to relieve the pain caused by PD. This treatment is almost widely used in all dynasties of China.^[23]^ With more and more modern research on moxibustion, some studies have shown that Moxibustion at 2 acupoints (CV4 and CV8) achieve the significant effect in treatment and prevention of PD, it is available for the overwhelming majority cases of PD, except the one differentiated as the obstruction by damp, heat and stasis.^[24]^ Moxibustion has existed and been used throughout China for thousands of years. Due to its port ability and practicality in modern society, moxibustion has greatly promoted the research on treating for PD.^[15,25]^

### 1.3 Description of the objectives

This network meta-analysis (NMA) aims to evaluate the current evidence for the efficacy and safety of acupuncture and moxibustion therapies for the PD.

## 2 Methods and analysis

### 2.1 Study registration

The protocol of this network meta-analysis follows the recommendations of the Cochrane Handbook for Systematic Reviews of Interventions.^[26]^ This protocol was registered with the International Platform of Registered Systematic Review and Meta-Analysis Protocols (INPLASY) on 28 May 2020 and registration number is INPLASY2020500106. (https://inplasy.com/inplasy-2020-5-0106/).

### 2.2 Inclusion and exclusion criteria

Population, intervention, comparison, outcome and study (PICOS) strategy was employed.

#### 2.2.1 Types of study

For this study only RCTs for acupuncture and moxibustion in people with PD, published in English and Chinese, will be included. All RCTs published in English and Chinese about acupuncture and moxibustion for PD will be included.

#### 2.2.2 Type of participants

Study participants of different age ranges with all types of PD will be included, regardless of sex, race, occupation, education, nationality, etiology, and severity. Patients that were diagnosed with pelvic pathology or secondary dysmenorrhea will be excluded.

#### 2.2.3 Type of interventions

In the experimental group, acupuncture (it includes body acupuncture, electroacupuncture, fire acupuncture, acupoint injection, and ear acupuncture) or moxibustion (it includes suspended moxibustion, Thunder fire moxibustion, taiyi miraculous moxa roll, mild moxibustion, needle warming moxibustion) will be included as a single intervention or major part of a combination therapy.

#### 2.2.4 Type of comparator (s)/control

In the control group, the comparative interventions will be sham acupuncture, sham moxibustion, placebo, no treatment, herbal medicine, NSAIDs, or other active treatments.

#### 2.2.5 Types of outcome measurements

##### 2.2.5.1 Main outcomes

Main outcome indicators: the extent of pain in the lower abdomen measured by visual analog scale (VAS), and relief from symptoms.

##### 2.2.5.2 Additional outcomes

Additional outcomes include the following aspects:

1. Quality of life (QoL);
2. Adverse events.

### 2.3 Database and search strategies

We will electronically search the following databases for literature, regardless of publication status and language: the Cochrane Central Register of Controlled Trials (CENTRAL); PubMed; EMBASE; China National Knowledge Infrastructure (CNKI); Chinese Biomedical Literature Database (CBM); Chinese Scientific Journal Database (VIP database); and Wan-Fang Database (WF).Search terms include acupuncture(body acupuncture, electroacupuncture, fire acupuncture, acupoint injection, and ear acupuncture) and moxibustion (it includes suspended moxibustion, Thunder fire moxibustion, taiyi miraculous moxa roll, mild moxibustion, needle warming moxibustion) and (primary dysmenorrhea or dysmenorrhea or painful menstruation) and (randomized controlled trial). The search will be restricted to human subjects, while there is no restriction on any specific languages. The proposed search strategy for PubMed is presented in Table 1.

**Table 1:**
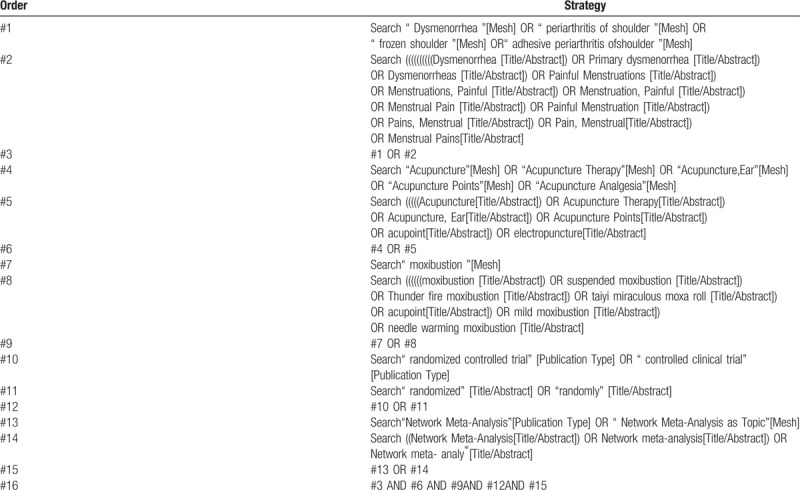
Search strategy (PubMed).

### 2.4 Studies selection

**Figure 1:**
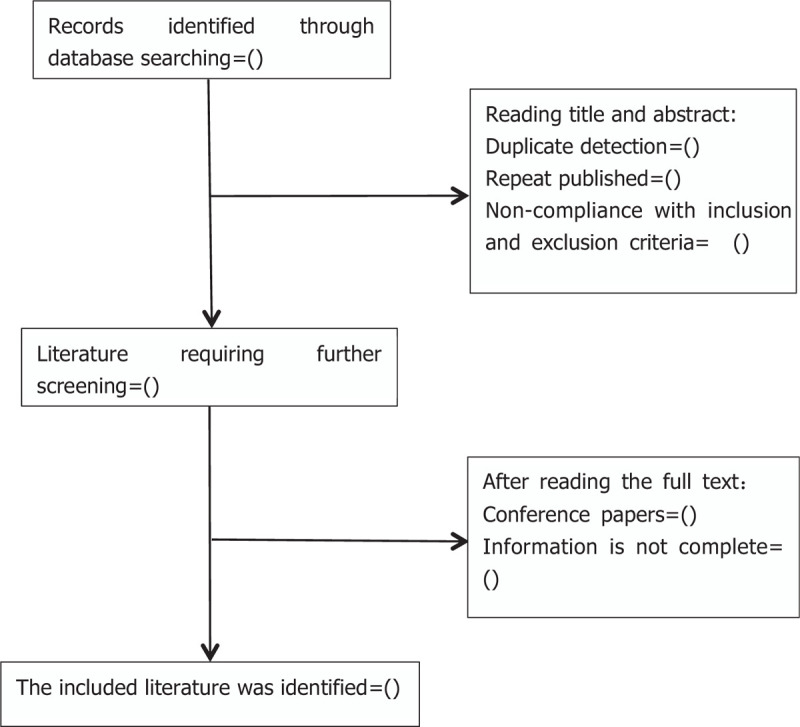
Flowchart of literature selection.

All studies identified through electronic and manual searches will be identified using NoteExpress (v3.2.0.7103). The process for identification and selection of studies will be conducted in 2 separate steps:

1. After the initial removal of duplicate studies, two reviewers (ZNW and YXY) will independently screen titles and abstracts based on the eligibility criteria. Full-text studies will be retrieved for all potentially includable SRs or SR protocols.
2. If studies contain insufficient information to make a decision about eligibility, one of the researchers (QSX) will try to contact authors of the original reports to obtain further details. During the procedure, disagreements will be resolved by discussion or consensus with the third reviewer (YY). Study selection will be performed in accordance with the PRISMA^[27]^ flowchart (Fig. 1).

### 2.5 Data extraction

Two independent researchers (ZNW and YY) use a pretested and standardized Microsoft Excel data extraction form to extract the literature information according to the inclusion and exclusion criteria, including the following aspects:

(1) Study characteristics: title, first author, year of publication, journal, registration number to trials registries, country of conduct, number of study arms, method of randomization and blinding, country of conduct, number of centers, and funding/ sponsor.
(2) Patient characteristics: gender, age, sociodemographic characteristics, sample size, number randomized to each arm, duration of disease, disease diagnostic criteria, and type of PD;
(3) Intervention: intervention measures (the experimental group and the control group) included type of therapy, frequency, the course of treatment, and therapy setting.
(4) Outcome of the study: The outcome characteristics included number of events, mean and standard errors, or standard deviation per arm. Two researchers (YY and QSX) cross-examined the results of extraction of the included literature. In case of differences, a third party (JX) should be consulted to resolve them.

### 2.6 Assessment of risk of bias

Two of our researchers (YY and QSX) will use the bias risk tool provided by the Cochrane Collaboration to evaluate the quality of the literature.^[28]^ This recommended tool includes 7 important items: sequence generation, allocation concealment, blinding of participants and personnel, blinding of results evaluation, incomplete result data, selective result reporting, and other biases. Make “Low risk,” “High risk,” and “unclear risk” judgments for each research literature. Finally, a “risk of deviation” summary and a chart is generated to show the results. As with the previous process, it will be independently assessed by 2 researchers (JX and ZNW).If there is disagreement, it will be discussed with the 3rd researcher(ZYZ).

### 2.7 Data analysis

#### 2.7.1 Characteristics of the eligible studies

Descriptive statistics will be conducted and the population characteristics of the eligible studies will include following items: age, duration of disease, types of comparisons, duration of study, methodological variables and country, and so on.

#### 2.7.2 Analytical procedure

To be able to compare active treatment with the same intervention with different controls, we will conduct a series of pairwise meta-analyses. The Bayesian network meta-analysis will evaluate the relevant results by directly and indirectly comparing acupuncture or moxibustion with control conditions.

#### 2.7.3 Pairwise meta-analyses

We will perform pairwise meta-analyses for each pairwise comparison to obtain odds ratios for dichotomous outcomes or standardized mean differences (SMD) for continuous outcomes, both with 95% credible intervals (CrIs). The heterogeneity of each pairwise comparison will be tested by *x*^2^ test (test level *a* = 0.1), a fixed effect model was used for meta-analysis of the results. If statistical heterogeneity exists among the results (*P* < .1, *I*^2^ > 50%), the source of heterogeneity needs to be analyzed. If there were significant heterogeneity between a group of studies, we would explore the reasons for the existence of heterogeneity from various aspects. When there is too much heterogeneity among the results of various studies, statistical methods such as subgroup analysis, sensitivity analysis and descriptive analysis can be used to treat the heterogeneity.

#### 2.7.4 Network meta-analyses

The Markov chain Monte Carlo method in WinBUGS 1.4.3^[29]^ will be used perform network meta-analysis (NMA) to synthesize direct and indirect evidence. The evidence relationship incorporated into the study will be calculated by STATA 14.0.^[30]^ The selection of the final model will depend on the deviance information criterion (DIC) value. Generally, a model with a smaller DIC value is better. We will use odds ratio with 95% CrIs to report the results of dichotomous outcomes, whereas for continuous variables, we will use mean difference with 95% CrIs to assess the effect size. The ranking analysis of NMA can estimate the ranking probabilities of all treatments for each outcome. After comparing multiple interventions, we will calculate the surface under the cumulative ranking curve (SUCRA).^[31]^ The greater the SUCRA value, the greater the probability of becoming the best intervention.

#### 2.7.5 Assessment of inconsistency

The difference between direct comparative evidence and indirect comparative evidence can be assessed by Consistency hypothesis, and it plays an important role in the NMA. When a loop is established among interventions, we can use *Z* test to assess the inconsistency.

#### 2.7.6 Heterogeneity, subgroup analysis, and sensitivity analysis

The heterogeneity between trials is quantified with the *I*^2^ and *P* values. When *P* < .1, *I*^*2*^ > 50%, no heterogeneity is considered among the trials, and the fixed effects model will be used for statistical analysis; otherwise, the random effects model will be used. When the clinical heterogeneity between the two studies is large, only descriptive analysis will be undertaken.

If the necessary data are available, we will conduct subgroup analyses to assess heterogeneity according to the different control measures (e.g., Western medicine, no treatment, placebo acupuncture or placebo moxibustion, etc), period of treatment, and outcome measures.

#### 2.7.7 Assessment of reporting bias

The publication bias will be assessed using a funnel plot when the included systematic reviews per outcome (RQLQ, VAS, and side effects) is more than 10.

#### 2.7.8 Evaluating the quality of the evidence

After the evidence is evaluated by the Grading of Recommendations Assessment, Development and Evaluation (GRADE), the quality of the evidence, the weights of strengths and weaknesses, and the patient's values and preferences will be carefully considered by 2 authors (ZNW and YXY) to develop preliminary recommendations. This proposal will be reviewed by 2 to 3 rounds of Delphi process,^[32]^ which will then be submitted to other 2 authors (YY and ZYZ) for approval. We will use the GRADE Grid instrument^[33]^ to review each recommendation one by one to group it into one of five options, including “strong recommendation,” “weak recommendation,” “unclear recommendation,” “weak no recommendation,” and “strong no recommendation”. The aim is to reach a better consensus. If 75 percent of the experts agree on an option, there is consensus on the recommendation. Otherwise, the project goes to the next Delphi process to discuss the disputed project again. We will evaluate the quality of treatment effect estimates from NMA by the following 4 steps:

1. Present acupuncture and moxibustion treatment estimates for each comparison of the evidence network.
2. Rate the quality of each acupuncture and moxibustion effect estimate.
3. Present the NMA estimate for each comparison of the evidence.
4. Rate the quality of each NMA effect estimate.

### 2.8 Ethics and dissemination

The study will not contain any personal data and will not prejudice individual rights, so no ethical approval will be required. The study will be subject to rigorous peer review and may be published in a journal or circulated at relevant conferences.

## 3 Discussion

We have developed this study based on the principles and standards of evidence-based medicine, in collaboration with multidisciplinary experts, which will facilitate the treatment of PD by clinicians, as well as for teaching and educating patients.

Based on what we know so far, we have found some limitations of this Protocol:

(1) Most of the sites included in the study are in China;
(2) The application of acupuncture and moxibustion in other countries and regions needs further study.
(3) At the same time, the differences of acupuncture and moxibustion operation methods between different countries should also be considered.

This study has a number of Contributions:

1. to the best of our knowledge, this is the first protocol to assess acupuncture and moxibustion therapy for patients with PD;
2. the results of this protocol will be beneficial to acupuncturists and physicians to make decisions the optimal method of treating the disease, and help patients with PD seeking optimal treatment;
3. the results are helpful to find out the correct operation method of acupuncture and moxibustion for treating PD and the relationship between the therapeutic effect, the time of acupuncture and moxibustion and the total amount of acupuncture and moxibustion, effectively improving the efficacy and safety of PD with acupuncture and moxibustion.

## Author contributions

**Conceptualization:** Yi Yang, Jun Xiong, ZhengYun Zuo.

**Data curation:** Yi Yang, XinYu Yu, ZeNanWu.

**Formal analysis:** XinYu Yu, QingShan Xie.

**Investigation:** Jun Xiong, Yi Yang.

**Methodology:** Jun Xiong, XinYu Yu, ZeNan Wu.

**Software:** Yi Yang, QingShan Xie.

**Supervision:**Jun Xiong, ZhengYun Zuo.

**Writing – original draft:** Jun Xiong, Yi Yang, XinYu Yu, ZeNan Wu.

**Writing – review & editing:** ZhengYun Zuo, XinYu Yu, QingShan Xie.
